# COVID-19 in trauma: a propensity-matched analysis of COVID and non-COVID trauma patients

**DOI:** 10.1007/s00068-021-01699-9

**Published:** 2021-05-25

**Authors:** Eric O. Yeates, Areg Grigorian, Morgan Schellenberg, Natthida Owattanapanich, Galinos Barmparas, Daniel Margulies, Catherine Juillard, Kent Garber, Henry Cryer, Areti Tillou, Sigrid Burruss, Ryan Arthur Figueras, Georgi Mladenov, Megan Brenner, Christopher Firek, Todd Costantini, Jarrett Santorelli, Terry Curry, Diane Wintz, Walter L. Biffl, Kathryn B. Schaffer, Thomas K. Duncan, Casey Barbaro, Graal Diaz, Arianne Johnson, Justine Chinn, Ariana Naaseh, Amanda Leung, Christina Grabar, Jeffry Nahmias

**Affiliations:** 1 Department of Surgery, University of California, Irvine (UCI), 333 The City Blvd West, Suite 1600, Orange, CA 92868-3298 USA; 2 Department of Surgery, University of Southern California (USC), Los Angeles, CA USA; 3 Department of Surgery, Cedars-Sinai Medical Center, Los Angeles, CA USA; 4 Department of Surgery, University of California, Los Angeles (UCLA), Los Angeles, CA USA; 5 Department of Surgery, Loma Linda University, Loma Linda, CA USA; 6 Department of Surgery, University of California, Riverside/Riverside University Health System, Moreno Valley, CA USA; 7 Comparative Effectiveness and Clinical Outcomes Research Center (CECORC), Riverside University Health System, Moreno Valley, CA USA; 8 Department of Surgery, University of California, San Diego (UCSD), San Diego, CA USA; 9 Department of Surgery, Sharp Memorial Hospital, San Diego, CA USA; 10 Trauma Department, Scripps Memorial Hospital La Jolla, La Jolla, San Diego, CA USA; 11 Department of Surgery, Ventura County Medical Center, Ventura, CA USA; 12 Santa Barbara Cottage Hospital, Cottage Health Research Institute, Santa Barbara, CA USA

**Keywords:** COVID-19, Coronavirus, Trauma, Mortality, Pneumonia, Length of stay

## Abstract

**Purpose:**

There is mounting evidence that surgical patients with COVID-19 have higher morbidity and mortality than patients without COVID-19. Infection is prevalent amongst the trauma population, but any effect of COVID-19 on trauma patients is unknown. We aimed to evaluate the effect of COVID-19 on a trauma population, hypothesizing increased mortality and pulmonary complications for COVID-19-positive (COVID) trauma patients compared to propensity-matched COVID-19-negative (non-COVID) patients.

**Methods:**

A retrospective analysis of trauma patients presenting to 11 Level-I and II trauma centers in California between 1/1/2019–6/30/2019 and 1/1/2020–6/30/2020 was performed. A 1:2 propensity score model was used to match COVID to non-COVID trauma patients using age, blunt/penetrating mechanism, injury severity score, Glasgow Coma Scale score, systolic blood pressure, respiratory rate, and heart rate. Outcomes were compared between the two groups.

**Results:**

A total of 20,448 trauma patients were identified during the study period. 53 COVID trauma patients were matched with 106 non-COVID trauma patients. COVID patients had higher rates of mortality (9.4% vs 1.9%, *p* = 0.029) and pneumonia (7.5% vs. 0.0%, *p* = 0.011), as well as a longer mean length of stay (LOS) (7.47 vs 3.28 days, *p* < 0.001) and intensive care unit LOS (1.40 vs 0.80 days, *p* = 0.008), compared to non-COVID patients.

**Conclusion:**

This multicenter retrospective study found increased rates of mortality and pneumonia, as well as a longer LOS, for COVID trauma patients compared to a propensity-matched cohort of non-COVID patients. Further studies are warranted to validate these findings and to elucidate the underlying pathways responsible for higher mortality in COVID trauma patients.

## Introduction

The Severe Acute Respiratory Syndrome Coronavirus 2 (SARS-CoV-2) causing the COVID-19 pandemic has infected millions, resulting in respiratory failure and multiorgan injury throughout the world [1–4]. In response, impressive progress has been made in understanding the unique pathophysiology and multisystem manifestations of COVID-19 [5–11]. However, many questions remain regarding which populations are most at risk for complications from this novel disease [12–14].

There is mounting evidence that surgical patients with COVID-19 have higher morbidity and mortality than patients without COVID-19 [15–17]. These outcomes are hypothesized to be associated with the stresses of surgery and mechanical ventilation in the setting of concurrent infection [16]. We suspect traumatic injury to similarly trigger poor outcomes; however, this has not yet been described in trauma patients.

As the COVID-19 pandemic continues and infection remains prevalent, we aim to evaluate the effects of COVID-19 status on morbidity and mortality in a multicenter trauma population [18]. We hypothesize that COVID-19-positive (COVID) trauma patients will have a higher mortality, longer length of stay (LOS), and increased pulmonary complications (i.e., pneumonia and ventilator days) compared to similar propensity-matched COVID-19-negative (non-COVID) patients.

## Methods

A retrospective analysis of trauma patients presenting to 11 American College of Surgeons (ACS) Level-I and II trauma centers in Southern California between 1/1/2019–6/30/2019 and 1/1/2020–6/30/2020 was performed. Though COVID-19 testing started in February 2020, we used a larger time frame utilized by a prior study to provide additional controls needed for propensity matching. This study was approved by the University of California, Irvine and each participating site’s Institutional Review Board and deemed exempt from need for consent.

All patients in each institution’s trauma registry were included, which contained both trauma activations and trauma consults. The primary outcome was in-hospital mortality. Secondary outcomes were length of stay (LOS), intensive care unit (ICU) LOS, mechanical ventilation, ventilator days, pneumonia, and ventilator-associated pneumonia (VAP). Demographic data and medical history were collected which included sex (self-reported), age, race (self-reported), insurance status, comorbidities, and body mass index (BMI). Injury characteristics and clinical data were collected and included blunt/penetrating mechanism, specific injury mechanism, blood alcohol level, urine toxicology, injury severity scores (ISS), abbreviated injury scale (AIS) scores for all body regions, and vital signs on arrival. COVID-19 testing results, related symptoms (fever, cough, shortness of breath, chills, body aches, nausea/vomiting, fatigue, sore throat, loss of taste/smell, headache, diarrhea), and presence of ground glass opacities on chest imaging were also collected [1, 2, 4]. COVID-19 testing practices and the exact laboratory test varied over time and by institution. Across centers selective screening for COVID-19 started as early as 2/25/2020 to as late as 4/8/2020 with the majority of centers starting in mid-March. Universal screening of all trauma patients started in the majority of centers in mid to late April. Patients were considered COVID-19 positive (COVID) if they tested positive within five days of admission to include only patients infected around the time of their injury. Additional outcomes collected included operations performed, complications, and discharge disposition.

Due to the observed imbalance in sample size, we used a 1:2 propensity score model to match COVID patients to non-COVID patients. Variables used in matching were reached by consensus of co-authors and included proven predictors of mortality in the trauma population such as age, blunt/penetrating mechanism, Glasgow Coma Scale score, systolic blood pressure, respiratory rate, heart rate, and ISS [19, 20]. Cases within 0.0001 of the estimated logit were included [21]. No COVID patients were excluded. Descriptive statistics were performed for all variables within each group. Categorical variables were reported as the number of occurrences with percentages of their respective group and continuous variables reported as means with standard deviations. Mann–Whitney-U tests were used to compare continuous variables and chi-square used to compare categorical variables. All *p* values were two-sided and considered significant if less than 0.05. This analysis was performed using IBM SPSS Statistics for Windows (Version 24, IBM Corp., Armonk, NY).

## Results

### COVID-19-positive trauma patients and symptoms

A total of 20,448 trauma patients were identified. 53 were COVID patients, accounting for 0.8% of patients presenting after 3/19/2020. Fifteen (28.3%) patients had any COVID-19-related symptom on arrival and the most common symptoms were fever (17.0%), cough (9.4%), shortness of breath (5.7%), and diarrhea (5.7%) (Table 1). Additionally, 14 patients (26.4%) had ground glass opacities on chest imaging.

**Table. 1:** Symptoms on admission for COVID-19-positive trauma patients

Symptoms, *n* (%)	*n* = 53
Any of the following symptoms	15 (28.3%)
Cough	5 (9.4%)
Shortness of breath	3 (5.7%)
Diarrhea	3 (5.7%)
Chills	1 (1.9%)
Aches	1 (1.9%)
Nausea/vomiting	1 (1.9%)
Fatigue	1 (1.9%)
Headache	1 (1.9%)
Sore throat	0 (0.0%)
Loss of taste/smell	0 (0.0%)

### Demographics and comorbidities

Compared to 106 non-COVID patients, the 53 COVID patients were more likely to be uninsured (11.3% vs 1.9%, *p* = 0.010) and have congestive heart failure (CHF) (3.8% vs. 0.0%, *p* = 0.044), but less likely to be of Asian race (0.0% vs 8.5%, *p* = 0.029) and have private insurance (22.6% vs 40.6%, *p* = 0.025). Otherwise, the two groups were similar with regards to sex, age, comorbidities, and BMI (all *p* > 0.05) (Table 2).

**Table. 2:** Demographics and comorbidities of propensity-matched COVID-19-negative vs COVID-19-positive trauma patients

Characteristic	COVID-19 negative	COVID-19 positive	*p* value
	(*n* = 106)	(*n* = 53)	
Male, *n* (%)	63 (59.4%)	36 (67.9%)	0.298
Age, years, mean ± sd	43.03 ± 22.98	45.34 ± 24.36	0.632
Race, *n* (%)			
White	44 (41.5%)	23 (43.4%)	0.820
Black	6 (5.7%)	1 (1.9%)	0.274
Asian	9 (8.5%)*	0 (0.0%)	0.029*
American Indian	0 (0.0%)	0 (0.0%)	n/a
Native Hawaiian	0 (0.0%)	0 (0.0%)	n/a
Latino	39 (36.8%)	25 (47.2%)	0.208
Middle Eastern	0 (0.0%)	0 (0.0%)	n/a
Insurance status, *n* (%)			
Medicare	17 (16.0%)	13 (24.5%)	0.197
Medicaid	41 (38.7%)	22 (41.5%)	0.731
Private	43 (40.6%)*	12 (22.6%)	0.025*
Uninsured	2 (1.9%)	6 (11.3%)*	0.010*
Comorbidities, *n* (%)			
Diabetes	6 (5.7%)	7 (13.2%)	0.102
Congestive heart failure	0 (0.0%)	2 (3.8%)*	0.044*
Cerebrovascular accident	3 (2.8%)	0 (0.0%)	0.216
Myocardial Infarction	0 (0.0%)	0 (0.0%)	n/a
Coronary artery disease	0 (0.0%)	0 (0.0%)	n/a
Cancer	0 (0.0%)	0 (0.0%)	n/a
End-stage renal disease	0 (0.0%)	1 (1.9%)	0.156
COPD	4 (3.8%)	3 (5.7%)	0.585
Dementia	5 (4.7%)	6 (11.3%)	0.122
Cirrhosis	0 (0.0%)	0 (0.0%)	n/a
Current smoker	11 (10.4%)	9 (17.0%)	0.237
BMI, mean ± sd	26.63 ± 5.72	27.82 ± 5.88	0.210

*Sd* standard deviation, *COPD* chronic obstructive pulmonary disease, *BMI* body mass index

*values are significantly different

### Injury characteristics and clinical data

Compared to non-COVID patients, COVID patients were less likely to test positive for alcohol (17.0% vs 45.3%, *p* < 0.001). Otherwise, the two groups were similar with regards to injury mechanism, urine toxicology, ISS, AIS scores, and vital signs on arrival (all *p* > 0.05) (Table 3).

**Table. 3:** Injury characteristics and clinical data of propensity-matched COVID-19-negative vs COVID-19-positive trauma patients

Characteristic	COVID-19 negative	COVID-19 positive	*p* value
	(*n* = 106)	(*n* = 53)	
Mechanism of injury, *n* (%)			
Blunt	91 (85.8%)	45 (84.9%)	0.873
Ground level fall	26 (24.5%)	16 (30.2%)	0.445
Fall from height	5 (4.7%)	4 (7.5%)	0.467
Pedestrian struck	8 (7.5%)	3 (5.7%)	0.659
Motorcycle collision	5 (4.7%)	2 (3.8%)	0.785
Motor vehicle collision	30 (28.3%)	9 (17.0%)	0.118
Assault	12 (11.3%)	8 (15.1%)	0.499
Sports injury	1 (0.9%)	3 (5.7%)	0.073
Penetrating	15 (14.2%)	8 (15.1%)	0.873
Gunshot	3 (2.8%)	4 (7.5%)	0.172
Stab wound	10 (9.4%)	3 (5.7%)	0.413
Alcohol positive, n (%)	48 (45.3%)*	9 (17.0%)	< 0.001*
Urine toxicology, n (%)			
Amphetamines	13 (12.3%)	7 (13.2%)	0.866
Barbiturates	0 (0.0%)	0 (0.0%)	n/a
Benzodiazepines	2 (1.9%)	1 (1.9%)	1.000
Opioids	5 (4.7%)	1 (1.9%)	0.377
Cocaine	6 (5.7%)	1 (1.9%)	0.274
PCP	0 (0.0%)	0 (0.0%)	n/a
THC	15 (14.2%)	5 (9.4%)	0.398
MDMA	1 (0.9%)	1 (1.9%)	0.615
ISS, mean ± sd	6.19 ± 6.59	7.55 ± 8.41	0.774
AIS Head/Neck	0.80 ± 1.17	0.75 ± 1.33	0.538
AIS Face	0.30 ± 0.62	0.49 ± 0.75	0.101
AIS Chest	0.60 ± 1.14	0.62 ± 1.20	0.930
AIS Abdomen	0.12 ± 0.41	0.28 ± 0.82	0.400
AIS Spine	0.17 ± 0.61	0.04 ± 0.28	0.144
AIS Extremity	0.63 ± 0.99	0.62 ± 0.93	0.861
AIS External	0.41 ± 0.49	0.38 ± 0.53	0.632
ISS > 16, *n* (%)	7 (6.6%)	8 (15.1%)	0.084
Vitals on arrival, mean ± sd			
Systolic blood pressure	134.59 ± 20.59	136.09 ± 26.31	0.518
< 100 mmHg, *n* (%)	2 (1.9%)	3 (5.7%)	0.199
Respiratory rate	19.03 ± 3.84	19.66 ± 4.64	0.332
Heart rate	90.72 ± 22.16	91.13 ± 22.09	0.864
Temperature, Fahrenheit	98.16 ± 1.37	98.65 ± 1.01	0.158
GCS score	13.85 ± 2.74	13.74 ± 2.67	0.586
< 13, *n* (%)	12 (11.3%)	8 (15.1%)	0.499

*ISS* injury severity score, *sd* standard deviation, *AIS* abbreviated injury scale, *GCS* Glasgow Coma Scale

*values are significantly different

### Outcomes

Compared to non-COVID patients, COVID patients had a longer mean LOS (7.47 vs 3.28 days, p < 0.001), ICU LOS (1.40 vs 0.80 days, *p* = 0.008), and a higher rate of pneumonia (7.5% vs. 0.0%, *p* = 0.011) and mortality (9.4% vs 1.9%, *p* = 0.029). Otherwise, the two groups had similar rates of mechanical ventilation, ventilator days, operations, complications, and discharge disposition (all *p* > 0.05) (Table 4). On detailed chart review by site investigators of COVID patient mortalities, it was reported that two COVID patients (3.8%) died of COVID-19-related respiratory failure. COVID may have contributed to an additional two patients but was not the direct cause of death (i.e., a patient who underwent palliative extubation after a high spinal cord injury and a patient with B-cell acute lymphatic leukemia with blast crisis). One likely non-related COVID death was due to a devastating brain injury.

**Table. 4:** Outcomes of propensity-matched COVID-19-negative vs COVID-19-positive trauma patients

Outcome	COVID-19 negative	COVID-19 positive	*p* value
	(*n* = 106)	(*n* = 53)	
LOS, days, mean ± sd	3.28 ± 7.04	7.47 ± 7.71*	< 0.001*
ICU LOS, days, mean ± sd	0.80 ± 2.34	1.40 ± 2.33*	0.008*
Mechanical ventilation, *n* (%)	10 (9.4%)	8 (15.1%)	0.288
Ventilator, days, mean ± sd	0.37 ± 1.89	0.47 ± 1.53	0.297
Operations, *n* (%)			
Tracheostomy	2 (1.9%)	1 (1.9%)	1.000
Laparotomy	2 (1.9%)	1 (1.9%)	1.000
Craniectomy/craniotomy	0 (0.0%)	0 (0.0%)	n/a
Vascular/endovascular	1 (0.9%)	1 (1.9%)	0.615
Complications, *n* (%)			
Sepsis	0 (0.0%)	1 (1.9%)	0.156
Stroke	0 (0.0%)	0 (0.0%)	n/a
Myocardial infarction	0 (0.0%)	1 (1.9%)	0.156
Pneumonia	0 (0.0%)	4 (7.5%)*	0.011*
Ventilator-associated pneumonia	0 (0.0%)	0 (0.0%)	n/a
Acute kidney injury	0 (0.0%)	1 (1.9%)	0.156
Acute renal failure	0 (0.0%)	0 (0.0%)	n/a
Deep venous thrombosis	1 (0.9%)	0 (0.0%)	0.478
Pulmonary embolism	0 (0.0%)	1 (1.9%)	0.156
Delirium tremens	0 (0.0%)	0 (0.0%)	n/a
Discharge disposition, *n* (%)			
Home	71 (67.0%)	32 (60.4%)	0.411
Skilled nursing facility	9 (8.5%)	6 (11.3%)	0.565
Long-term acute care hospital	0 (0.0%)	1 (1.9%)	0.156
Acute rehabilitation	4 (3.8%)	0 (0.0%)	0.152
Hospice	0 (0.0%)	0 (0.0%)	n/a
Mortality, *n* (%)	2 (1.9%)	5 (9.4%)*	0.029*

*LOS* length of stay, *ICU* intensive care unit, *sd* standard deviation

*values are significantly different

## Discussion

The social, economic, and mortality effects of COVID-19 have been widespread and pronounced throughout society. This multicenter retrospective study of trauma patients demonstrated a nearly fivefold higher rate of mortality, higher pneumonia rate, and a longer LOS and ICU LOS for COVID trauma patients compared to a similarly matched non-COVID trauma cohort.

To our knowledge, this is the first study to demonstrate an increase in mortality for COVID trauma patients. In many respects, our findings are consistent with accumulating reports regarding increased morbidity and mortality for COVID surgical patients [15–17]. For example, Doglietto et al. found an approximately eightfold higher mortality rate in COVID surgical patients, similar to this study’s fivefold increase [15]. The worse outcomes in these studies predominantly were attributed to pulmonary and thrombotic complications stemming from both the pro-inflammatory and immunosuppressive responses to surgery in the setting of COVID-19 infection [15, 16]. We suspect a similar pathophysiologic response to trauma and report an increase in pneumonia rates and two patients who died, at least in part, due to COVID-19 related respiratory failure. However, our reported pneumonia rates are much lower than previous studies on surgical patients and we did not observe an increase in ventilator-associated pneumonia, deep venous thrombosis, pulmonary embolism, or the need for mechanical ventilation in our COVID cohort [15–17]. This absence of the full-spectrum of COVID-19-related complications in this study is likely multifactorial. First, many of the COVID patients likely recovered from previous COVID or had only mild infection, which is supported by our low rates of COVID-19-related symptoms and imaging findings [22, 23]. Second, the low ISS scores in our study indicate that a large portion of our COVID cohort sustained minor trauma which perhaps is not a powerful enough insult to trigger COVID-19 complications. COVID trauma patients merit additional confirmatory studies and basic science research to further determine the exact areas of disruption to coagulation and inflammatory pathways that could potentially identify at-risk patients or mitigate this risk [6].

Predicting LOS in the trauma population is vital to informing management of care, resource utilization, and throughput for high-volume trauma centers [24]. This study identified an increase in both total LOS and ICU LOS for COVID trauma patients, which has not yet been described in the literature. We believe the longer ICU LOS likely reflects that COVID patients were more critically ill due to COVID-19 itself as they had a similar ISS compared to the non-COVID cohorts. The increase in total LOS is likely similarly an effect of the more ill COVID patients; however, it could also be related to placement difficulties leading to a prolonged hospital stay [25]. This longer LOS and ICU LOS should be noted by trauma surgeons and administrators as the pandemic continues and a higher rate of COVID occurs in the community and hospital beds become increasingly scarce.

There are multiple limitations to our study. First, our data relied on multiple trauma registries and data collectors, making the process vulnerable to miscoding and misclassification. Second, though we attempted to include all COVID trauma patients presenting during the study period, likely some were missed early in the pandemic when routine testing of all trauma patients had not begun. Third, we could have matched patients based on additional characteristics like race, insurance status, and comorbidities, which may have made our groups more similar. Unfortunately, this would have led to unmatched COVID patients, which we felt was not acceptable with an already small sample, which is in and of itself a limitation to this study. Notably, our COVID cohort was more likely to have CHF and be uninsured, both of which are known risk factors for mortality in trauma [26–29]. Additionally, COVID patients were less likely to be alcohol positive, though we doubt alcohol contributed to increased mortality as its relationship to mortality in trauma is controversial and some studies have even suggested it improves mortality [30–36]. Our COVID cohort also had a higher ISS and a higher percentage of patients with hypotension, though this did not reach statistical significance. Also, only two patients in the COVID group definitively died of COVID-19-related complications. Finally, we did not have access to inflammatory markers, coagulation disturbances, or exact timing of COVID-19 symptoms, which would have provided additional insight into the severity of COVID-19 infection, which may have been predominantly minor infections in our study population given there was no difference in rate of pneumonia. Future studies should consider stratifying COVID patients by infection severity and including a more injured population of COVID-positive trauma patients to allow for a more complete description of the virus’ effects on trauma outcomes.

## Conclusions

This multicenter retrospective study of 11 ACS Level-I and -II trauma centers found increased rates of mortality and pneumonia, as well as a longer LOS, for COVID trauma patients compared to a similar propensity-matched cohort of non-COVID patients. Further studies are warranted to validate these findings and elucidate the underlying cause (e.g., worsened respiratory failure), as well as predisposing factors responsible for higher mortality in COVID trauma patients.

## Data Availability

Raw data can be made available upon request.
